# Anaphylaxis to Oral Trimethoprim-Sulfamethoxazole in a Child: A Case Report

**DOI:** 10.7759/cureus.79317

**Published:** 2025-02-19

**Authors:** Haruna Mabuchi, Naoki Kajita, Go Kusakawa, Kumiko Morita, Koichi Yoshida

**Affiliations:** 1 Division of Allergy, Tokyo Metropolitan Children's Medical Center, Fuchu, JPN; 2 Department of Pediatrics, National Defense Medical College Hospital, Tokorozawa, JPN

**Keywords:** basophil activation test, drug allergy, drug provocation test, pediatric drug hypersensitivity, trimethoprim-sulfamethoxazole (tmp-smx)

## Abstract

Trimethoprim-sulfamethoxazole (TMP-SMX) is a non-β-lactam antibiotic commonly used in pediatrics to treat infections and as a prophylactic medication. Hypersensitivity to TMP-SMX is generally non-immediate, and immediate allergic reactions, including anaphylaxis, are rare. This case report details a six-year-old girl who experienced anaphylaxis to TMP-SMX. Her skin prick test with TMP-SMX was negative, but she developed anaphylaxis in a drug provocation test (DPT). An additional basophil activation test (BAT) for the TMP-SMX combination tablet was evaluated and was confirmed positive. This case underscores the need for alternative diagnostic methods like BAT, which pose a lower risk than DPT. The findings suggest that BAT could offer a safer diagnostic approach, though more studies are required to validate its use for TMP-SMX allergy diagnosis.

## Introduction

Trimethoprim-sulfamethoxazole (TMP-SMX) is a combination of two antimicrobial agents: a diaminopyrimidine (TMP) and a sulfonamide (SMX) [1]. In pediatrics, TMP-SMX is used to treat acute infections such as urinary tract infections and skin infections caused by methicillin-resistant *Staphylococcus aureus* (MRSA). It is also used as prophylaxis for pneumocystis pneumonia in immunocompromised patients [2,3]. While TMP-SMX is generally well tolerated, it does have several adverse effects. Dermatologic conditions are the most common, including maculopapular eruption, erythema multiforme, toxic epidermal necrolysis, and Stevens-Johnson syndrome [2,4]. Anaphylactic reactions, although rare, can occur [4,5]. Herein, we describe a pediatric case of anaphylaxis to TMP-SMX confirmed by a drug provocation test (DPT). In preparing this case report, we explained this report to her guardian and obtained consent. 

This case was previously presented as a meeting abstract at the 73rd Annual Meeting of the Japanese Society of Allergy on October 19, 2024.

## Case presentation

A six-year-old girl had recurrent skin and soft tissue infections such as furuncle and folliculitis since the age of four. She was referred to our infectious diseases department for treatment at the age of four years and nine months because a wound culture revealed MRSA. After that, she took TMP-SMX for treatment, and by the time she was six years old, she had repeated skin infections at least four times. About six months after the last treatment of furuncle, she again experienced swelling with redness around her eye. She took the oral TMP-SMX, and generalized hives and dyspnea appeared after 10 minutes of injection. Two weeks later, similar skin symptoms appeared around her eyes. She took the oral TMP-SMX again, and 10 minutes later, generalized hives and cough appeared. She visited the emergency room during both of those two episodes, but on evaluation in the emergency room, her vitals were normal, she was clinically stable, and she was discharged without any intervention. Other medical history included allergic rhinitis, which she treated with nasal corticosteroids. She also occasionally took oral antihistamines for her allergic rhinitis symptoms. There was no other family history of allergic predisposition, except that her mother had atopic dermatitis.

**Figure 1:**
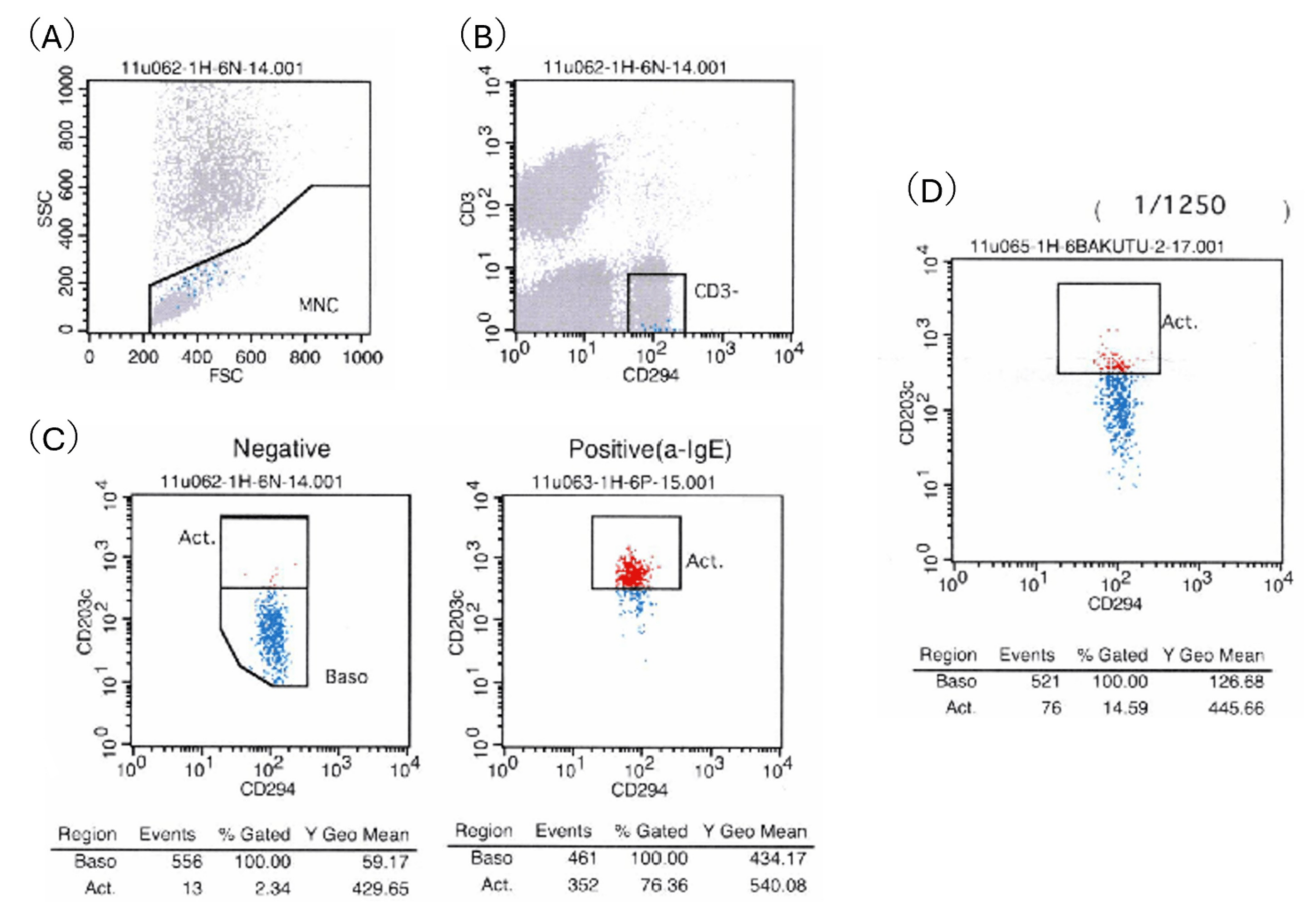
Basophil activation test for TMP-SMX combination tablets (TMP 80 mg/SMX 400 mg) (A) MNCs were identified in whole blood as SSC low and FSC. (B) Basophils were identified in MNCs as CD3-/CD294+ cells. (C) Negative and positive control. The test reagent for the negative control is RPMI 1640 medium (with 10% ABS), and for the positive control, it is a-IgE. (D) 1/1250 tablet concentration. Activated basophils are expressed as the percentage of CD203c+ cells in the Act. gate. TMP: trimethoprim; SMX: sulfamethoxazole; MNCs: mononuclear cells; Act.: activation; SSC: side scatter; FSC: forward scatter; RPMI: Roswell Park Memorial Institute; ABS: human AB serum; a-IgE: anti-IgE

At her first visit to the allergy department, she had a single furuncle on her left shoulder but was not taking any antimicrobial medication. A skin prick test (SPT) was performed at concentrations of SMX 0.8 mg/mL and TMP 0.16 mg/mL [6], with a negative result. An intradermal test (IDT) was not performed as consent was not obtained. The reaction of SPT was negative, and since immediate reactions to TMP-SMX are rare, we planned DPT to confirm the negative result. DPT was scheduled as an open challenge with an oral TMP-SMX tablet (SMX 400 mg/TMP 80 mg) and was performed after obtaining written parental informed consent. Fifty minutes after administration, frequent coughing (Grade 3 of respiratory tract symptoms on Sampson's Symptom Grade), erythema, and pruritus on the trunk and upper limbs appeared (Grade 2 of the skin). She was administered an antihistamine tablet, but oral pruritus (Grade 1 of the gastrointestinal tract) appeared after administration. All symptoms resolved within 60 minutes. The positive DPT result confirmed that she had an immediate allergy to TMP-SMX. Since the SPT was negative, the basophil activation test (BAT) was evaluated as a biomarker for the presence or absence of allergic conditions in the future. By evaluating BAT, we also evaluated whether IgE-mediated mechanisms were involved. As a result, basophil activation was 14.59% (0.32 mg/mL of SMX and 0.064 mg/mL of TMP being>10% considered positive). The positive cutoff values were set based on past data from the commissioned inspection company. The negative and positive controls were 2.3% and 76.4%, respectively (Figure 1). Regarding the suspicion of additive hypersensitivity, Bacta tablets (Shionogi Pharmaceuticals Co., Ltd., Osaka Japan) contain the additives hydroxypropyl cellulose, magnesium stearate, and carboxymethylcellulose calcium. The patient had been taking other medications containing these additives before and after the onset of symptoms. An allergy to TMP-SMX was suspected, but there were no particular allergic symptoms to those medications. Therefore, we concluded that the additives were not the cause of the allergic reaction in this case.

## Discussion

Patients experiencing a sulfonamide antimicrobial allergy may experience a variety of clinical manifestations. These may include hypersensitivity reactions from each of the Gell and Coombs classification. Although less common than other hypersensitivity reactions, type 1 IgE-mediated reactions result in manifestations such as anaphylaxis [7]. We report a case of anaphylaxis to TMP-SMX as shown by DPT.

In a review of anaphylactic cases using the Japanese Adverse Drug Event Report database, which has more than 500,000 reports from 2004 through 2018, antibiotics were the cause in about 12% of cases, with β-lactams accounting for 80% of these cases [8]. TMP-SMX was not mentioned in the review. In children with drug hypersensitivity confirmed by diagnostic allergy tests in Turkey, only one TMP-SMX allergy case was found among 168 drug allergy children diagnosed based on SPT and/or DPT [9]. It is not stated whether the TMP-SMX allergy case was immediate- or delayed-type, nor did it describe how the diagnosis was made in detail. In a report from Italy, six of the 637 children suspected of drug allergy, whether immediate or non-immediate, were thought to be caused by TMP-SMX based on clinical history [10]. Two of them had a history of immediate-type symptoms, but only one presented a positive SPT result, and in neither case was the diagnosis confirmed by DPT.

According to the guidelines and practical parameters for drug hypersensitivity reactions [11,12], diagnosis work-up for immediate-type is based primarily on SPTs, IDTs, and DPTs. SPTs are less risky compared to IDTs and DPTs and are often conducted first, but they are less sensitive than the latter. The standard approach is to perform an IDT if the SPT is negative, followed by a stepwise DPT. In a report examining the safety of a single loading test in cases in which the symptoms of the suspected allergic reaction were not severe and more than five years had passed since onset, the test could be performed without serious adverse events [13]. Although this patient had been symptomatic for less than one year, we did not strongly suspect TMP-SMX as the causative agent and performed a single loading test because the SPT was negative and the frequency of TMP-SMX allergy in children is low. During the DPT, severe allergic symptoms were observed despite the negative SPT.

Based on the course of this case, even if the SPT is negative and the suspected agent is rarely reported, we believe that, for safety reasons, a DPT with divided doses should be considered. On the other hand, BAT has recently been considered as an in vitro test for immediate allergy using peripheral blood basophils [14]. Although BAT is still an investigational testing method, it has the advantage of being safe compared to in vivo tests. In a previous adult case with anaphylaxis to TMP-SMX [15], the BAT results for TMP-SMX and TMP alone were positive. The report also confirmed that basophils did not respond in the four negative subjects. SPT and DPT could not be performed due to a lack of the patient's consent, but TMP was diagnosed as the causative agent based on the BAT result.

The BAT result was also positive in the present case, and these findings suggest that immediate-type TMP-SMX allergy with anaphylaxis involves an IgE-dependent pathogenesis [14]. In a study of patients with suspected immediate allergy to amoxicillin or clavulanate [16], BAT was performed with each drug, with a positive predictive value of more than 90%. Another study examined the utility of BAT for drugs other than antibiotics and found a positive predictive value of 94.3% and a negative predictive value of 95.2% [17]. For antibiotic allergies other than β-lactams, the usefulness of BAT has also been reported in quinolone antimicrobial allergy [18]. Although the sensitivity and specificity of the BAT for TMP-SMX allergy are still difficult to evaluate due to the paucity of data, it may become a useful test in the future as more cases are accumulated. 

## Conclusions

We describe a rare case of anaphylaxis due to TMP-SMX, which was confirmed by a DPT and supported by a positive BAT result. This case underscores the importance of considering TMP-SMX allergy even when immediate hypersensitivity reactions are rare. While DPT provides a definitive diagnosis, it carries a significant risk of severe allergic reactions. Therefore, for methods such as the development and validation of a safer diagnostic, it might be beneficial to establish a method for the clinical application of BAT and to evaluate a concentration that increases the sensitivity of SPT. These alternative methods may offer a safer approach for diagnosing TMP-SMX allergy, reducing the need for high-risk procedures such as DPT.
